# Adverse event reporting and patient safety: the role of a just culture

**DOI:** 10.3389/frhs.2025.1581516

**Published:** 2025-08-20

**Authors:** Augustine Kumah

**Affiliations:** ^1^ Quality Department, The Bank Hospital, Accra, Ghana; ^2^ Policy & Research Department, Research on Interventions for Global Health Trabsformation – RIGHT Institute, Accra, Ghana

**Keywords:** adverse event, a just culture, incident reporting, patient safety, quality care

## Abstract

Reporting adverse events is essential for ensuring patient safety and fostering a culture of continuous improvement in healthcare. Adverse events, defined as unintended injuries or complications arising from healthcare management, offer crucial insights into systemic weaknesses that, if addressed, can prevent future harm. However, underreporting such events remains a significant challenge, often driven by fear of punitive actions, reputational damage or legal repercussions. To address these concerns and promote a robust reporting culture, healthcare organisations must adopt a just culture by implementing standardised frameworks for evaluating errors and establishing robust reporting systems. A culture that emphasises accountability and learning over punitive measures. Leadership commitment, psychological safety, and fair accountability are foundational to fostering a just culture in healthcare. While each theme presents specific requirements and challenges, their integration is essential for building a resilient and learning-oriented healthcare system.

## Introduction

Patient safety is a cornerstone of high-quality healthcare, yet adverse events—unexpected incidents that result in harm to patients—continue to pose significant challenges (1, 2). Effective adverse event reporting is essential for identifying risks, understanding system vulnerabilities, and preventing future harm. However, fear of punishment or blame often discourages healthcare professionals from reporting errors and near-misses, leading to missed opportunities for improvement (1–3). This reluctance highlights the need for a Just Culture, an approach that balances accountability with a focus on learning and improvement rather than punishment.

A Just Culture fosters an environment where healthcare workers feel safe reporting errors without fear of retribution (1, 4, 5). It distinguishes between human mistakes, at-risk behaviors, and reckless actions, ensuring that responses to adverse events are fair and constructive. By prioritizing learning over punishment, organizations can uncover the root causes of incidents, implement effective safety interventions, and create a culture of continuous improvement (6).

Learning from errors is vital for all organizations, as it involves examining incidents or near misses to uncover their root causes and generate knowledge that can guide future practices. This process is essential in building safer, more reliable, and resilient systems (7, 8). Rather than focusing solely on enforcing rules or addressing the actions of individual employees—known as compliance strategies, many scholars emphasize that mistakes often stem from broader structural and institutional issues (7–9). Therefore, a comprehensive approach is needed, one that fosters an ethical organizational climate and promotes collective responsibility among staff members.

In the healthcare sector, enhancing patient safety is frequently linked to the ability to learn from past incidents. A central concept in this effort is the creation of a “just culture,” which refers to organizational practices that aim to reach fair outcomes for individuals involved in adverse events or near misses (5, 9, 10). At the core of a just culture is a shift away from blame and punishment toward openness, healing, and organizational learning (4, 10). Leading researchers have argued that retributive justice—where the emphasis is on penalizing individuals for errors—undermines learning and safety (1, 7, 10). Instead, they advocate for restorative justice, which focuses on repairing harm and supporting the well-being of everyone affected by an incident (10).

Restorative justice within a just culture encourages a workplace environment where staff feel empowered to speak up about issues, not only after problems arise but also proactively to enhance care delivery (9–12). It emphasizes collaboration, dialogue, and mutual understanding, aiming to improve systems rather than punish individuals (4, 10, 12). The goal is to promote transparency, trust, and continuous improvement, rather than fear and silence.

However, despite the positive intentions behind the just culture model, debates persist regarding its effectiveness in practice. Critics question whether such frameworks consistently lead to fair treatment and genuine cultural change (9, 11, 13). Findings from organizational research highlight that cultivating a strong safety culture is a multifaceted and often difficult process, with no guaranteed success (9, 11). Many organizations struggle to translate the theoretical benefits of a just culture into practical and sustainable outcomes (14, 15).

This discussion underscores the significance of learning from adverse events and the foundational principles of a just culture. It also stresses the importance of embedding this philosophy into healthcare systems through intentional strategies. Doing so requires leadership support, structured processes for incident reporting and analysis, and a commitment to continuous learning and ethical accountability. Building a culture where staff feel psychologically safe to report concerns, learn from mistakes, and contribute to improvement is essential for enhancing the overall quality and safety of care (7, 9, 14, 15).

Creating a learning-oriented and just organizational culture demands more than procedural reforms. It involves a fundamental transformation in how errors are perceived and addressed, prioritizing growth, healing, and fairness over punishment. While challenges remain, particularly in aligning ideals with outcomes, the pursuit of a just culture is a critical step toward safer and more responsive healthcare systems. This perspective explores the importance of adverse event reporting, the principles of a Just Culture, and strategies for integrating this approach into healthcare organizations.

## Methods

This perspective article employed a non-systematic search and thematic content analytical approach to explore the systemic factors influencing adverse event reporting in healthcare, with a specific focus on the role of a Just Culture. The objective was to explore the importance of adverse event reporting, the principles of a Just Culture, and strategies for integrating this approach into healthcare organizations.

A non-systematic search of the literature published between 2015 and 2024 was conducted across multiple databases, including PubMed, Scopus, and Google Scholar. Search terms included “adverse event reporting,” “Just Culture,” “patient safety,” “non-punitive systems,” “healthcare leadership,” “reporting behavior,” and “Strategies.” Articles published between 2015 and 2025 were considered to ensure the inclusion of contemporary perspectives and practices. National policy documents, reports and strategy documents on implementing a just culture were also reviewed.

## Results

A literature search reveals three main themes which are important for fostering a just culture in healthcare organizations: leadership commitment, open communication, psychological safety, and balanced accountability. Table 1 below summarizes the three main themes essential for fostering a just culture in healthcare organizations. It outlines the key features and requirements of each theme, their contributions to promoting a just culture, and common challenges associated with their implementation.

**Table 1:** Themes for fostering a just culture in healthcare organizations.

Theme	Key features/requirements	Purpose/contribution to just culture	Challenges/considerations
Leadership commitment	- Demonstrates fairness, transparency, and learning - Models openness and supports incident reporting - Invests in system-level improvements and staff support	- Sets the tone for organizational values - Drives sustained culture change - Ensures consistent and fair responses	- Lack of visible leadership engagement can undermine trust - Competing priorities may hinder investment in safety culture
Open communication & psychological safety	- Fosters a non-punitive environment - Ensures staff feel safe to report errors - Maintains feedback loops and transparent communication	- Encourages error reporting and learning - Builds trust among staff - Enhances reflection and shared problem-solving	- Fear of blame or retaliation may persist - Requires consistent reinforcement and visible responsiveness to staff feedback
Balanced accountability	- Differentiates between human error, at-risk, and reckless behavior - Uses system redesign, coaching, or discipline appropriately - Applies consistent standards	- Promotes fairness while maintaining responsibility - Reduces fear and encourages reporting - Aligns justice with learning goals	- Misjudging behaviors can lead to unfair consequences - Needs careful implementation to maintain credibility and fairness

## Discussion

### Barriers to adverse event reporting

Despite its potential, the implementation of just culture faces several obstacles. A predominant challenge is the deeply ingrained blame culture within many healthcare organisations (1, 16, 17). Historical reliance on punitive measures has created an environment where professionals fear repercussions, discouraging transparency (1, 16, 17). Additionally, managerial inconsistency in addressing errors often undermines trust in the system (17). For instance, discrepancies in how similar incidents are handled can create perceptions of unfairness, further discouraging reporting (1, 17).

Another barrier is the lack of understanding and awareness of just culture principles among healthcare staff (1, 2, 16, 17). Without proper training and education, employees may misinterpret the approach as being lenient or as failing to hold individuals accountable (1). Legal and regulatory pressures also pose challenges, as concerns about litigation can deter organisations from fully embracing non-punitive reporting frameworks (1, 17).

### Themes for fostering a just culture in healthcare organizations

#### Leadership commitment

Leadership plays a pivotal role in creating and sustaining a just culture. Leaders must actively demonstrate a commitment to fairness, transparency, and learning, setting the tone for the organisation's values and expectations. Their behaviour influences how staff perceive safety culture and whether they feel supported in reporting incidents (9, 11). Effective leaders model openness, respond consistently to safety concerns, and invest in system-level improvements rather than resorting to individual blame (7, 18). Leadership engagement is also essential in allocating resources for education, staff support systems, and continuous quality improvement initiatives (7, 15). Without visible and sustained leadership commitment, efforts to establish a just culture often falter (11, 15, 19).

#### Open communication and psychological safety

Creating an environment where healthcare workers feel psychologically safe to speak up about errors or near misses is crucial to fostering a just culture. Psychological safety refers to the belief that individuals can report mistakes or express concerns without fear of punishment or humiliation (7, 12, 18). It encourages learning from incidents by promoting open dialogue and reflection, thus contributing to better patient safety outcomes (4, 7, 15, 20). Organisations must establish non-punitive reporting systems and ensure that feedback loops are maintained to show staff that their reports lead to meaningful change (4, 5). Transparent communication also helps demystify the process of incident investigations, fostering trust and engagement (4, 5).

#### Balanced accountability

A just culture does not eliminate accountability; instead, it differentiates between human error, at-risk behaviour, and reckless behaviour. Human error is managed through system redesign, while at-risk behaviour is addressed with coaching and behavioural reinforcement (15, 18). Reckless behaviour, which demonstrates a conscious disregard of substantial risk, may warrant disciplinary action (3). By applying consistent and fair responses based on the nature of behaviour rather than outcomes alone, healthcare organisations can build a culture that is just and conducive to improvement (3, 9, 14). This nuanced approach supports individual responsibility while recognizing the role of organisational systems in shaping behaviour (9, 14, 18).

### Implementing “A just culture”

Implementing a just culture in healthcare requires a multifaceted approach that addresses organisational, managerial and individual factors. Leadership commitment is paramount; leaders must model just cultural behaviours, demonstrate accountability and prioritise safety over blame (2, 16, 17). Developing clear policies and guidelines for error classification and response is equally important as it ensures consistency and fairness in how incidents are addressed (3).

Education and training programmes are vital in promoting awareness and understanding of just culture principles (1, 17). These programmes should emphasise the distinction between human errors, at-risk behaviours and reckless conduct, providing staff with the tools to respond appropriately. Role-playing scenarios, workshops and case studies can help reinforce these concepts and demonstrate their practical application (3).

The integration of non-punitive reporting systems is another critical component. Such systems should be designed to facilitate easy and confidential reporting, with mechanisms to protect the anonymity of reporters when appropriate. Feedback loops are essential for ensuring that staff are informed about the outcomes of reported incidents, which can reinforce the value of reporting and build trust in the system (1, 3, 17).

**Figure 1:**
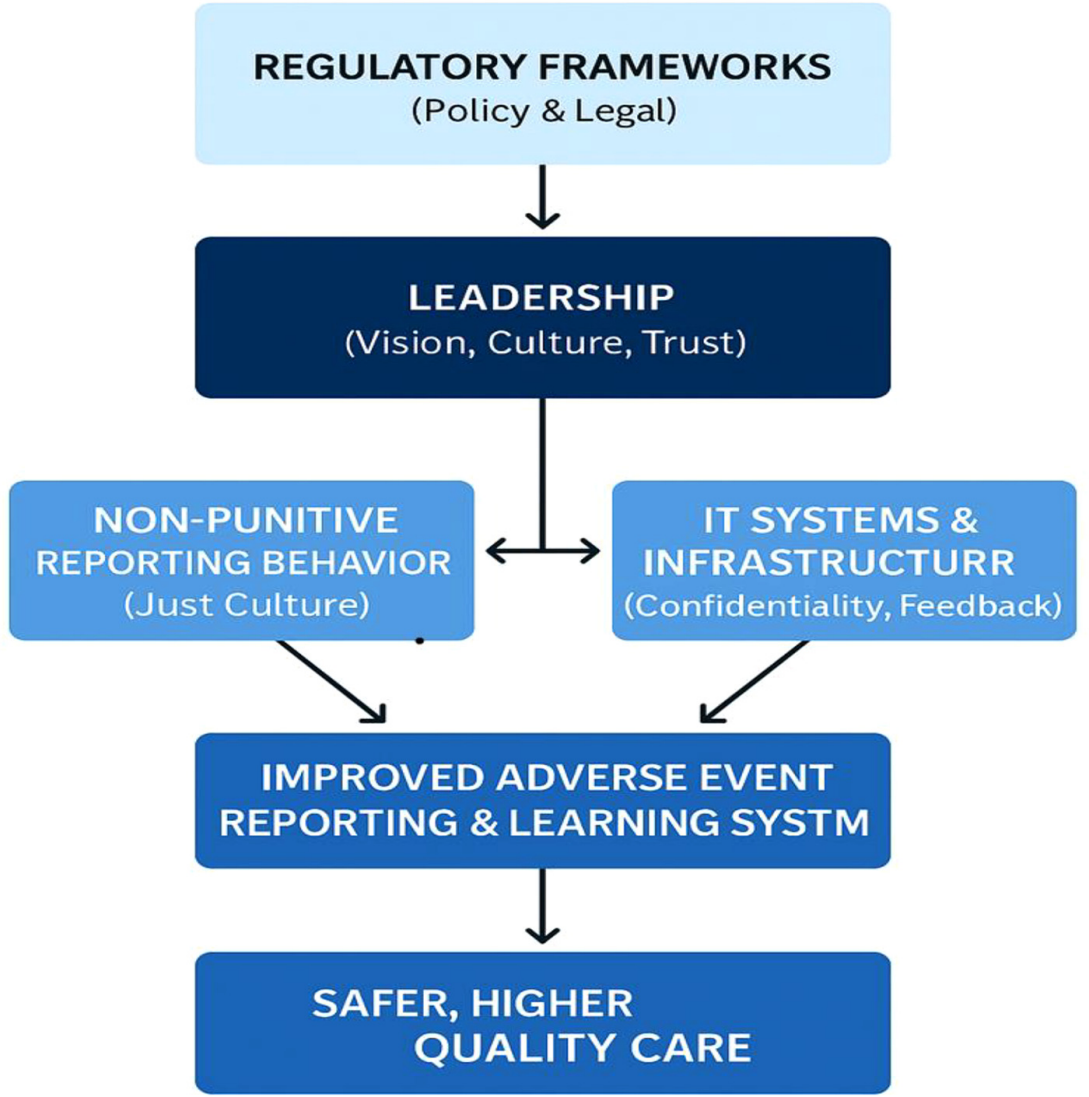
Systemic interdependencies for adverse event reporting and patient safety.

Regulatory Frameworks (Figure 1) set the tone for legal protections, reporting mandates, and liability boundaries. When they promote non-punitive policies, they empower leadership to implement Just Culture principles confidently (3, 4, 15, 18). Leadership translates regulatory intent into practice by building trust, ensuring fairness in incident response and allocating resources for IT and training. Also, leadership directly influences both reporting behavior through modelling, psychological safety, supporting non-punitive processes and IT systems by investing in platforms that ensure confidentiality, ease of use, and feedback. Reporting behavior is shaped by the perceived fairness and safety within the organization (4). It feeds critical data into IT Systems, which enables incident tracking, trend analysis, and transparent feedback loops. These elements converge to produce a robust learning system that identifies risks, implements systemic improvements, and reduces future harm, ultimately leading to improved patient safety.

### Measuring the impact of just culture

Assessing the effectiveness of just culture initiatives requires developing standardised metrics and evaluation tools (1, 3). Key performance indicators may include reporting rates, staff perceptions of psychological safety and the frequency of systemic improvements resulting from reported incidents. Periodic surveys and interviews can provide valuable insights into staff attitudes and identify areas for improvement (17).

Case studies from organisations that have successfully implemented just culture can also serve as benchmarks for best practices. For instance, hospitals that report significant increases in adverse event reporting rates following the adoption of just culture principles often attribute their success to strong leadership, comprehensive training, and consistent application of policies (1, 3, 16, 17, 21).

### Sustaining cultural change

Sustaining a just culture requires ongoing commitment and adaptability (22). Organisations must regularly evaluate their policies and practices to ensure alignment with just culture principles. Staff feedback should be actively sought and incorporated into decision-making processes, fostering a sense of ownership and engagement (1, 3, 4, 22).

Continuous education and training are essential for reinforcing just culture behaviours and addressing emerging challenges (22). Additionally, leadership succession planning should prioritise candidates who are committed to upholding just culture principles, ensuring continuity in organisational values.

## Conclusion

Adverse event reporting is a fundamental component of patient safety, and the principles of just culture provide a robust framework for enhancing reporting rates and fostering systemic improvements. Fostering a just culture in healthcare requires a multi-faceted approach rooted in leadership commitment, the cultivation of psychological safety through open communication, and a fair and consistent system of accountability. These themes are interdependent and must be embedded into the fabric of the organisation to ensure that safety and learning are prioritized over blame and fear.

A just culture represents a paradigm shift in addressing adverse events, emphasising systemic improvement over individual blame. Its successful adoption has the potential to transform healthcare organisations, making them safer and more resilient. Future research should focus on developing standardized metrics to evaluate just culture initiatives and exploring their applicability across diverse healthcare settings.

## Data Availability

The original contributions presented in the study are included in the article/Supplementary Material, further inquiries can be directed to the corresponding author.
